# Worldviews: overarching concept, discrete body of knowledge or paradigmatic tool?

**DOI:** 10.1007/s40839-020-00113-7

**Published:** 2020-09-22

**Authors:** Ruth Flanagan

**Affiliations:** University of Exeter, Exeter, UK

**Keywords:** Worldviews, Teachers, Religious Education

## Abstract

The term ‘worldviews’ is employed across disciplinary boundaries, yet with no agreed definition it may actually obscure rather than clarify meaning. The use of the term has grown in frequency, particularly in Religious Education (RE) in England, since the Commission on RE’s final report (2018), which recommended changing the name of RE to ‘Religion and worldviews’. Response to the report has been mixed. Some fear that an introduction of worldviews may lead to a dilution of RE and overburden an already overstretched teaching profession; others welcome a meaningful study of non-religious worldviews which they view as more pertinent in the current growth of ‘nones’ (Nones’ term used for those who adhere to ‘no faith’, see Woodhead (J Br Acad 4:245–261, 2016)) in England. Teaching worldviews raises questions of selection: are all worldviews equally appropriate for pupils to study and consistent with the aims of education? For example, is it appropriate for a 6 year old to study Hedonism or National Socialism? This paper problematizes the binary nature of the debate and interrogates the usage of the term 'worldviews'. Focusing on ‘institutional worldviews’ is questionable as the role of master narratives, embedded in these, lose currency. The ‘disintegration of master narratives’, (Riessman in Narrative Methods for the Human Sciences. SAGE Publications, Thousand Oaks, 2008, p. 17), has led to a rise in individuals creating their personal embodied worldviews, albeit subconsciously. Rather than consider worldviews as a discrete body of knowledge that imposes on an already overburdened curriculum, I propose that employing worldviews as an overarching concept, providing a type of paradigmatic analysis for RE, may lead to a greater and more profound understanding of religion(s).

## Introduction

The term ‘Weltanshcauung’ or worldview has evolved from its philosophical roots in Kant’s (1790) initial use to infiltrate a range of disciplines including philosophy (Hegel 1807; Dilthey 1907), sociology (Walsh and Middleton 1984), anthropology, history, theology (Naugle 2002; Duderija 2007), and psychology (Freud 1933, 1938; Jung 1942). Within each discipline, differing definitions and employment of the term have evolved which, even within the same discipline, can be contradictory: within Psychology, for example, Freud (1938) deems worldviews as static, yet Jung (1942) saw his comparable ‘philosophy of life’ as elastic and flexible.

## The English context

The term has recently become more dominant in the field of RE in England, not least with the Commission on RE’s (2018) recommendation to change the name of RE to ‘Religion and worldviews’. The CoRE (2018) report has faced criticisms with some fearful that the addition of worldviews risks creating too thin an RE curriculum with too much to cover. In a letter to the Religious Education Council of England and Wales, Damian Hinds (2018), the then Secretary of State for Education, rejected the Commission’s proposals stating that “some stakeholders” had expressed “concerns that making statutory the inclusion of ‘worldviews’ risks diluting the teaching of RE”. To teach six major religions and then to teach a range of organised worldviews as well would be onerous for teachers already struggling to cover the RE curriculum in the limited time set by many schools for RE (Barnes 2015). Smalley, as chair of NASACRE,^1^ queried the usefulness of the name change and called for a greater academic debate over which worldviews were deemed worthy of study (2018). Barnes (2015) previously queried whether extending the curriculum in RE to include humanism and non-religious worldviews was necessary educationally and questioned whether this may lead to reinforcement of stereotypes and a potential lack of depth. Yet, as trained professionals, teachers can mitigate against this. However, is this an unnecessary imposition on their time?

Whether the Government will accept the recommendations is unclear and, in light of Brexit and the Coronavirus pandemic, decisions may be delayed. However, there are positive voices concerning the introduction of ‘worldviews’ in RE. The report builds on Dinham and Shaw’s (2015) recommendations for a more inclusive approach to RE which represents the real landscape. Postive responses include the creation of a Professor of ‘Religions and Worldviews’ at Canterbury University (2020) and Chater’s call to reform RE (2020) employing worldviews education. Yet confusion exists with the term worldview as this has often been employed to denote secular beliefs, such as in Norway. There is an inconsistency in language used at policy and classroom level (Oddrun and Everington 2019). In defence of the CoRE report Cooling (2020) acknowledged the messiness of the concept of worldviews, but recommended ‘as Freathy and John (2019) exhort…embracing the messiness to create workable curricula and resources’ (p. 10).

## Literature review: what are worldviews?

A search of relevant literature reveals the ‘messiness of worldviews’ (Cooling 2020), and current discussions on worldviews reflect, but have yet to resolve, historical debates on content or component dimensions, character, construction and definitions of the concept.

The literature refers to worldviews as discrete bodies of knowledge as ‘institutional’ worldviews (CoRE 2018, p. 4) or ‘organised’ worldviews (Van der Kooij et al. 2013). These are worldviews, such as humanism, secularism, capitalism, materialism, which have a recognised or agreed set of beliefs and values acknowledged by a group and often embedded in institutions or societies.

In contrast, worldviews defined as an overarching concept or framework for thought (Aerts et al. 2007) echoes Hegel’s (1807) view of worldviews as ‘conceptual frameworks’. Indeed, Hegel’s ‘conceptual frameworks’ have been influential in philosophical thought: ‘the notion of alternative *conceptual frameworks* has been a commonplace of our culture since Hegel’ (Rorty 1982, p. 3).

The question of *content* (2013)^2^ of worldviews contributes to the debate—are these static categories or fluid component dimensions (Koltko-Rivera 2004,3)? Suggestions in the literature include an explanation of the world, a futurology, values and answers to ethical issues, a praxeology, an epistemology and aetiology. Politics, religion, values and societal norms are all impacted by, and aspects of, individual’s worldviews. If religion(s) are an aspect of worldviews then they are not synonymous with worldviews. Therefore, ascribing ‘institutional’ worldview status to religion(s), or using the terms interchangeably, is problematic: the Christian worldview or the Islamic worldview. Indeed, Duderija (2007), challenges the idea of a single Islamic worldview and in attempting to delineate between differing Islamic worldviews, notes the key role that the interpretation of scripture plays in this process of differentiation. Similarly, Wolters (1985) attempted to differentiate between proposed ‘Christian’ worldviews by defining a Biblical worldview yet, rather than prove which one is *the* biblical worldview, his work actually demonstrates the existence of authentically held different interpretations of scripture.

Attributing ‘institutional’ worldview status to a religion may lead to stereotyping and less nuance whilst viewing religion as an aspect of a personal worldview may prove more useful. Indeed, Hegel’s (1807) reasoning on individual particularity provides foundational thought for this as he differentiated between a characteristic worldview that an individual may possess as well as a religiously informed one:

everyone may have his particular way of viewing things generally (weltanschauung) so he may have also a religion peculiar to himself (1807: 193).^4^

Thus, individuals may have worldviews that are informed by many different aspects: a ‘bricolage’ (Van der Kooji et al. 2013) of views.

The *character,* or nature, of worldviews is suggested as static when viewing worldviews as discrete bodies of knowledge, or ‘institutional’ worldviews (CoRE 2018, p. 4), reflecting Freud’s (1933) view of worldviews as static. Freud’s definition of worldviews was prompted by his rejection of attempts to define his form of psychoanalysis as a worldview. Freud deemed that labelling his work as a worldview presented unnecessary static restrictions on the dynamic nature of psychoanalysis. He rejected the term worldview rather than investigate the definition more closely. In contrast, Valk and Tosun (2016) deem the *character* of worldviews as dynamic and evolving, echoing Dilthey’s (1907/1958) metaphilosophy: a typology of worldviews with three interconnecting realms in dynamic relationship. Dilthey impacted the development of the theory of worldviews by devising a typology of worldviews—naturalism, idealism of freedom and objective idealism. For Dilthey worldviews are not static but rather the three types interact to facilitate revision. Naturalism, the world picture of reality, establishes the idealism of freedom, value of life. Objective idealism is the upper level of consciousness where the interpenetration of body, naturalism and mind, idealism of freedom occurs. The concept of dynamic, evolving worldviews find their birthplace in his work.

Additionally, is the *character* of worldviews a possession or a fundamental part of human life experience? Questions of whether all individuals hold worldviews (Hand’s ‘optionality’ 2012) reflects Kierkegaard’s (1843) assertion that not all have worldviews, or ‘life views’ as he describes them. Kierkegaard’s hierarchy of worldviews is evident as he views three distinct fields of aesthetic, ethical and religious, with religious placed at the summit. Cooling (2020) rejects the notion that individuals can ‘have’ a worldview but prefers to refer to individuals ‘inhabiting’ worldviews. Worldviews assisting an understanding of the inner self (Valk 2009) echoes Nietzsche’s (1887) search for the subterranean man. To understand individuals Nietzsche stressed the need to understand personal worldviews, which entailed seeing from another’s perspective. He employed the term worldviews but spent little time reflecting on the nature of worldviews except to deem them as a social construct.

Questions exist around the *Construction* of worldviews: constructed or evolved naturally in response to life experience? Is construction a conscious pursuit by individuals to solve ‘the riddle of life’ (Dilthey 1907) or unconscious (Sire 2004; Copley 2005). Hegel (1807) deemed worldviews as evolving throughout history, finding expression in art through propaganda, exhibition and propagation, moulded by and through the passing of time. Thus, an evolutionary socio-constructed path for personal worldviews.

A variety of metaphors have been posited to aid understanding of the term: sociologist Lappe and Lappe’s ‘map of the mind’ (2003, p. 9); in political science with Olsen’s system to guide its adherents through the social landscape (1992); in religious studies with Walsh and Middleton’s ‘model of the world which guides its adherents in the world’ (1984, p. 32); or in intercultural communication with Samovar and Porter’s ‘meaning overarching philosophy or outlook or concept of the world’ (2004, p. 103). These metaphors, whilst differing in nuance, provide support for worldviews as an overarching concept yet by no means conclude the debate. Valk (2009), whilst citing different metaphors provides a list, albeit not exhaustive, of differing codified worldviews: secular humanism, materialism etc.

Attempts to examine the concept have been undertaken by Apostel (1991), Aerts et al (1994), Naugle (2002) and Valk (2009). Aerts et al. (2007) building on Apostel’s extensive philosophical writing concluded that worldviews provided a frame of reference for individuals to interpret the world:

A world view is a system of co-ordinates or a frame of reference in which everything presented to us by our diverse experiences can be placed. It is a symbolic system of representation that allows us to integrate everything we know about the world and ourselves into a global picture, one that illuminates reality as it is presented to us within a certain culture (2007: 7).

Employing this definition implies that all individuals have worldviews developed from experiences which enable them to interpret themselves, others and the world around them. These may be formalised or informal and contain elements of both, therefore questioning the usefulness of a binary position.

Defining the concept may well be impacted by the concept, therefore adding to the complexity: Naugle (2002) and Sire (2004) point out that ‘the concept of worldviews is worldview dependent’ (Sire 2004, pp. 93–94). Naugle’s (2002) work led Sire to rethink his ‘inadequate definition’ (1988) to add the emotional sense. Schultz (2013) builds on their work to propose a 3D concept of worldviews: propositional, behavioural and heart-orientation. Sire claims that individuals ‘think not about one’s worldview but with it’ (2004, p. 124) which if so presents challenges for researchers and teachers of ‘Religion and worldviews’. These writers, whilst concerned primarily with research into a Christian worldview in the context of education in the USA, provide insight into the importance of an examination of the purposes of, or agenda for, employing the term and the benefits of a multifaceted approach to any attempt at definition.

The significant disparity within and between disciplinary definitions of the term worldviews allows for critical examination of the term. The messiness has led some to reject the usefulness of ‘worldviews’ within RE. For example, the recent Welsh consultation document for RE does not employ the term, preferring instead ‘Religion, Values and Ethics’ (2020).^5^ Yet to dismiss the term entirely may miss potential benefits.

For some worldviews provide a link for the growing number of nones in England (Woodhead 2016), and therefore this can assist in making RE relevant to all (Jones 2013). Other potential benefits include: to counter the challenge of religious illiteracy (Prothero 2007; Valk 2016); to aid meaningful interaction between differing worldviews (Mulder and Berg’s 2019, hermeneutical communicative model); to enhance global citizenship (Miedema and Bertram-Troost 2015); to challenge bigotry and intolerance (Barnes 2015); to assist integration into a multicultural society (Miller and Mckenna 2011); to prevent exclusivist teaching (Miedema 2014); to recognise the value laden-ness of all teaching (Miedema 2014, p. 94); and to focus more on views that sit well with the teachers’ own. Yet some face the charge, however ill founded, that in introducing worldviews they wish to dilute religions out of RE (Hinds 2018; Chater 2020).

An honest reflection on the agenda for employing the term may assist with unlocking these benefits. The strong connection between agendas and philosophy signifies the importance of this: ‘The agenda defines the range of problems and issues that are addressed by a philosophy’ (Vidal 2008: 3). Further examination of the practical challenges of implementing teaching worldviews may aid the debate.

## Challenge of teaching worldviews in practice

To define worldviews as discrete bodies of knowledge to teach in RE curriculums raises challenges of practicability.

Firstly, if teachers accept worldviews as discrete bodies of knowledge to be studied this raises the challenge over which should be chosen and by whom. Does Humanism, Paganism, Secularism, Communism, Nazism or Hedonism, for example, claim the greatest value in our curriculums and deserving of a place in our syllabus for ‘Religion and Worldviews’? The CoRE (2018) report delineates between ‘personal’ and ‘institutional’ worldviews and states that ‘institutional worldviews’ are to be studied. Whilst making passing reference to some worldviews, notably ‘Humanism, Secularism or Atheism’ (CoRE 2018, p. 26), it makes no explicit list of which institutional worldviews are deemed worthy of study, perhaps allowing local SACREs independence to choose those most relevant in their own localities. Barnes (2015) raises the question of which worldviews are relevant to study if worldviews are discrete bodies of knowledge. Yet this challenge alone is no reason to disregard worldviews but rather a need for clarity over value judgments in curriculum and syllabus design.

Secondly, approaches to worldviews education such as debating the merits of capitalism over communism, or vice versa, might be more suitably debated in Economics, History or Politics lessons than RE. Perhaps the question is of situating worldviews in the appropriate subjects and asking which are applicable, relevant or of value to RE. In a subject that has to vie for time on the timetable in many schools^6^ (CoRE 2018, p. 8) and teacher training courses (Ofsted^7^,^2013^), a clearer focus on the aspects of worldviews relevant to RE, rather than delving into the remit of other disciplines, seems more appropriate.

Thirdly, ‘worldviews’ can be perceived as an additional topic, with a body of knowledge with facts, dates, core beliefs and values, key individuals and institutions etc. for pupils to learn. This extra ‘topic’ may be added into RE as yet another area where the pupils may see no relevance to their own lives. Thus rather than assist RE to reach its potential (Ofsted 2013) this may provide barriers.

Fourthly, the issue of lack of time, even to cover the six major religions, so how can all worldviews be addressed as well – even if only a few are chosen? These practical challenges do not negate the fact that worldviews may indeed describe codified discrete bodies of knowledge, but rather they bring into question the usefulness and practicability of following such an approach in RE.

## Assistance in practice

Definitions of key terms evolve and adapt in an evolutionary process to suit the aims and purposes of their employer. What the RE world needs is not a definitive answer to the binary debate, of discrete body or overarching concept, but what is required is a way to improve the teaching and learning of RE in England. The initial aim of the CoRE (2018) noted that ‘it is impossible fully to understand the world without understanding worldviews—both religious and non-religious’, but acknowledged that ‘Religious Education (RE) in too many schools is not good enough to prepare pupils adequately for the religious and belief diversity they will encounter, nor to support them to engage deeply with the questions raised by the study of worldviews’ (2018, p. 3). To meet this aim I question whether studying a list of non-religious worldviews alongside religions will succeed but may instead lead to a dilution (Barnes 2015) through sheer volume encountered by teachers and pupils. I suggest employing Worldviews, with a capital W as an institutional/organised worldview and lower case ‘worldviews’ as embodied personal ‘lifeviews’ (Kierkergaard 1838). I propose that focusing on personal embodied worldviews provides a clear starting point for teachers and pupils to enter in to the subject of RE: to cross the divide between self and the perceived exotic or ‘other’ nature of religions.

Firstly, I suggest that the relevance and usefulness of studying Worldviews (organised, institutional worldviews) may have significantly reduced in the last 20 years. Globalisation, civil wars and increased immigration have undermined the usefulness of employing generic terms. This calls into question the usefulness of studying organised or institutional Worldviews. I am not employing the term Worldviews in place of religion(s), as previously mentioned. The narratives of religion(s) hold significant value in RE in explaining the history of religion(s) and the practices of their followers. When Nations or institutions undergo disrupting life experience such as civil war, global pandemics, mass immigration or migration the sense of common identity, or Worldview, may be shaken (Connor 1994; Weller and Wolff 2005). New forms of narrative that are more of an individual or much smaller community focus evolve. In today’s rapidly changing world, there are far fewer tightly knit communities, with codified Worldviews, which share similar experiences and have minimal external influences. Therefore, there may be less value in focusing on master narratives and a greater need to focus on the individual. The ‘disintegration of master narratives’, noted by Riessman (2008, p. 17), has led to a rise in individuals creating their own embodied worldviews, even if they often do so subconsciously. The ‘Bricolage’ concept (Van der Kooij et al 2013) of views collected by individuals throughout their lifetime and adopted as a part of their own worldviews does seem to resound with individuals’ life experiences.

Secondly, it is within these embodied worldviews and their formation that understanding of others can be assisted. Aerts et al.’s (2007) frame of reference provides a useful definition of what a worldview may entail. A more helpful metaphor to illustrate the nuanced and intricate nature of worldviews may be smart-tech eyewear. Employed in fictional books and films, such as the “Mission Impossible” franchise, smart-tech glasses contain the capability to perform real time cross-referencing against central databases. These provide information on individuals, corporations or situations that the wearer finds themselves. Glasses are a poor metaphor though as they can be removed whereas worldviews may not be so easily cast off. Another illustration is from the Terminator film (1984) where, in some scenes, the viewer sees the world through the robot’s eyes—through a red lens, which displays data and analysis, uploaded from his database, as he observes individuals and objects in his path. Similarly, worldviews provide individuals with information, data, about the situations they are in—people they meet, places they go, norms of practice, value of events/interactions/individuals etc.… Information is provided from a database—created from what the individual has read, or been taught through informal (community, family norms, media) or formal means (through schooling), or has experienced themselves. These databases are constantly updated with new information received through education or life experience. These experiences or information can then alter the individual’s worldview to a greater or lesser degree.

If Worldviews can be either discrete bodies or overarching concepts (Oddrun and Everington 2019), which is more useful for the improvement of the subject of RE; which assists pupils’ engagement and learning; which improves teachers’ understanding and confidence; which will equip pupils for life in a diverse world? Worldviews can refer to both, but I propose that the second may well be more useful in RE. Identifying and examining their own worldviews provides an effective picture of the greater interconnectedness of worldviews and their constantly evolving dynamic. Individuals can be made aware of the influences, which are constantly impacting their lives and their worldview.

Employing worldview as an overarching concept may prevent exclusivist claims: ‘all personhood education in schools is inherently worldview-laden, because it has to do with meaning-presenting, meaning-giving, meaning-making, meaning-taking and meaning in action’ (Miedema 2014, p. 94). To equip teachers and pupils to understand the existence of personal worldviews and the worldview laden-ness of their educational experience may assist them in their understanding of difference.

The implementation of worldviews as an overarching concept, rather than Worldviews as a discrete body of knowledge, provides opportunity for a type of paradigmatic analysis which may aid teaching and learning in RE.

## Employing worldviews as a type of paradigmatic analysis

Investigating personal worldviews may expedite and develop the future of Religious Education in England and, perhaps, in other countries also. The increasing numbers of individuals who adhere to ‘no faith’, has led to increasing numbers of teachers and pupils for whom religion and religious practice is alien or even viewed as exotic (Rollock 2009). As they begin to study religions and non-religious worldviews this can prove an obstacle as they attempt to seek out what is important or good to teach. RE is impacted by a wealth of choice on formal and informal levels through SACREs, examination boards, schools’ curriculum choices and individuals’ interpretation of these. RE can become a watered down version of the most palatable versions of the religion(s) or non-religious worldviews which adhere to the individuals’ personal worldviews (Flanagan 2019), or focus on the ‘brighter’ rather than the ‘darker’ side of religion(s) (Kueh 2017).

RE may remain on the surface and may miss the embedded meanings of these faiths. Learner-centred education, promoted by Dewey’s (1933) educational philosophy, influenced the Plowden report (1967), and led to a more child centric education, particularly at the primary level. The challenge for teachers of RE is how can they start from the child, if the child follows no particular faith. How can the pupils relate to a religious faith if they have no religious faith? What frame of reference can they employ?

This is where assistance may be provided by identifying and examining worldviews: becoming worldview conscious. All individuals have worldviews – a way that they see the world, which is held consciously and subconsciously (Sire 1988). If teachers and pupils can see that all individuals possess a worldview, then the study of religion(s) and non-religious worldviews becomes the study of fellow human beings and their worldviews—a search, which all have in common, to understand and make meaning in the world, whether or not that contains a religious or spiritual aspect. Worldviews become a paradigm—a model for human understanding. Worldview studies can become a means for conducting a type of paradigmatic analysis of religions. Rather than being in conflict, they work in concert. To understand worldviews provides a means for developing understanding of the embedded meanings of religion(s) and non-religious worldviews.

Paradigmatic analysis is a form of semiotic analysis that employs inductive and deductive means to identify common and contrasting themes between narratives.

‘Paradigmatic‐type narrative inquiry gathers stories for its data and uses paradigmatic analytic procedures to produce taxonomies and categories out of the common elements across the database’ (Polkinghorne 1995: 10).

Individuals’ life narratives can reveal the process of formation and evolution of personal worldviews. Examining these life narratives can be facilitated by paradigmatic analysis:

- to compare and contrast themes between narratives
- to uncover embedded meanings (Polkinghorne 1995).

Paradigmatic thought is employed to bring order to experience in the form of categories. Items, individuals or experiences are placed in categories, which are formed from previous experiences. Future experiences are then categorised alongside similar experiences. This echoes the formation of worldviews as a frame of reference. Worldviews are formed through paradigmatic reasoning, through the process of ordering and categorising experiences. Words are encoded and decoded, within appropriate cultural context, identifying patterns, with a basis of common understanding, to aid communication. With worldviews, miscommunication occurs not simply because people disagree but because their ‘paradigms are incongruent’ (Vroom 2006, p. x), so rather than common understanding their different valuations of rationality and criteria are the issue. Undertaking the process of identifying these paradigms, worldviews, may facilitate greater communication and understanding.

In RE teachers and pupils need

- to compare and contrast different religions and non-religious worldviews
- to uncover embedded meanings.

I propose that the process of personal worldview identification can facilitate a type of paradigmatic analysis: providing the means for RE teachers and pupils to compare and contrast different religion(s) and non-religious worldviews and assisting in uncovering embedded meanings. Examining their own worldviews enables individuals to have a basis from which to compare the worldviews of others. If pupils examine religions on a superficial level, such as issues of different dress for women, and impose their worldviews of women’s rights onto that religion, they may form a negative response to that religion. Without recognition of their own worldviews, and the fact that their own choice of clothing may well be informed by societal or cultural norms, this lesson may prove detrimental to their understanding of that religion. The differences they encounter may seem alien or even ‘wrong’, to them. To enable them to perceive that their understanding of ‘right’ and ‘wrong’ is part of their personal worldview, which has been informed through their life experience, can aid in this process. If they examine their own view of appropriate dress and decision making regarding their own clothes, trace influences on this and origins of values alongside their own social and cultural norms, they may then start to understand the valid existent of other differing, even opposite values and beliefs.

For example, why do some people feel it is acceptable to tell lies if they are being polite in doing so? This is because politeness may be valued more than total honesty. Why do many queue in an orderly way and get irate with others who do not? Why do I, as a teacher, think pupils who I am reprimanding should give me eye contact as a mark of respect and get annoyed when they do not? Yet in many cultures to look an authority figure in the eye is seen as disrespectful (Uono and Hietanen 2015). These very basic examples illustrate in a simplistic way the influence of worldviews from our social and cultural norms, which are subconsciously held but provoke hostile reaction when experiencing contrasting behaviours. This is exactly the type of situation that occurs in studying RE: religions are studied where the belief or practice of the faith is in direct opposition to the pupils own worldviews: role of women, dress, pursuit of holiness, LGBTQ+. Yet, other issues such as the golden rule or care for the poor are accepted and taught well as they fit with dominant views in the UK at the moment.

This is precisely where acknowledging and identifying the existence of personal worldviews and their impact can assist teachers in RE: rather than view religion(s) or non-religious worldviews as alien, unintelligible or even offensive they can become a valid response to shared life experience. In the 1960s Oberg employed the term ‘culture shock’ to explain the phenomenon which may occur when individuals live in a cross-cultural situation: “anxiety that results from losing all of our familiar signs and symbols of social intercourse” (1960, p. 177). This can result in frustration, anger, injury and disproval towards the unfamiliar culture. Later the term ‘learner shock’ was employed for the challenges that adults may face when returning to education after an absence:

experiences of acute frustration, confusion and anxiety experienced by some students (who) find themselves exposed to unfamiliar learning and teaching methods, bombarded by unexpected and disorienting cues and subject to ambiguous and conflicting expectations.

(Griffiths, Winstanley and Gabriel 2005: 275–276).

In RE teachers and pupils can face ‘worldview shock’ where they are confronted with worldviews that are opposed to their own, creating an emotional response of frustration, anger, injury or disproval for the unfamiliar worldview. To enable individuals to see that their worldview is exactly that, *their worldview,* and not the accepted ‘norm’ and only valid worldview for all people, is vital. Recently, a colleague was surprised to find that another colleague had voted in a different way from her on Brexit. The fact that this logical, rational and intelligent colleague could have such different worldviews on this issue shocked her.

Two main forms of research are possible with paradigmatic analysis:

1. where previous theory or logic is used to examine new data to see if these are found
2. where previous ideas are set aside to examine the data directly.

For this approach in RE these two forms are assisted by beginning with an examination of personal worldviews.

An example of the first type of paradigmatic analysis is the investigation into storied data and applying already known patterns for genres—categorising stories – for example, moral tales, historical stories or poetic writing. This first type may be demonstrated through the identification of an individual’s own worldviews, with its categories, and then examining similarities and differences between that and those of others: comparing and contrasting religion(s) and non-religious worldviews. Valk (2009) created a framework tool for worldview identification based on a fusion of current research and philosophical thinking on worldviews. This framework involves categorisation and was operationalised by Larkin et al. (2020). As teachers and pupils begin to examine their own (un)conscious worldviews and the specific categories contained within them, they may begin to understand more clearly worldviews that are different from their own. Rather than these appearing unusual, alien or exotic, they then become an intelligible response to shared life experiences. Those experiences can be examined in categories such as family, community, responsibilities, education, employment, values etc. For example, Valk employs a section on ultimate questions which provides a frame of categories:

1. What is the purpose of life?
2. How do I discern what is right/wrong?
3. How do I right my wrongs?
4. Do I have any responsibilities or obligations?
5. What happens when you die?
6. Is there a deity or greater force?

In lessons answering these ‘Ultimate questions’, or similar, for self and others can aid understanding and provide a framework for comparison. These have been critiqued in that many individuals never ask these questions nor have an answer to them or see the need for an answer to them (Hand 2012). Yet that does not negate the importance of the questions. Examining different questions that religion(s) and non-religious worldviews pose provides a useful entry and frame to examine similarities and differences between religion(s) and non-religious worldviews. Recognising that for some answering a question on ‘What is the purpose of life?’ is fundamental yet for others they see no need to even examine this, provides insight into embedded views beneath the surface of religion(s) and non-religious worldviews.

An example of the second type of paradigmatic analysis is where the researcher attempts to examine data without preconceived ideas or imposing categories on the data. This second type is the more challenging—involving the identification of an individual’s own views and a setting aside of these views to listen to the data itself. For RE teachers, this would entail an attempt to examine a religion or non-religious worldview whilst attempting to set aside the teacher’s or pupil’s own preconceived ideas—their worldviews.

## Conclusion

The recommendation to change the name of RE to ‘Religion and worldviews’ (CoRE 2018), whilst encountering some opposition, affords an opportunity to examine the teaching of RE. Rather than view worldviews as an additional body of knowledge for pupils to learn, teachers may employ the concept to enable pupils to connect with a subject that may, at first sight, appear irrelevant to their own lives. The two approaches, suggested in this article, demonstrate how employing worldviews as a type of paradigmatic analysis for RE may well facilitate a greater and more profound understanding of religion(s). Despite the messiness of the concept, worldview provides a model for human understanding, one that may prove vital for the future of RE.

## Footnotes

1 National Association of Standards Advisory Councils for Religious Education. In England, Standards Advisory Councils for Religious Education (SACREs) write locally agreed syllabi. Their members are comprised from local religious groups, county councillors, and teachers.

2 Questions of whether overarching content of narratives and truths exist led Hedlund-de-Witt to examine worldviews: ‘to fathom the complex, controversial, and much contemplated concept of worldviews’ (2013, p. 135).

3 Koltko-Rivera rejects the notion of categorical approaches for worldviews as they qualitatively categorise individuals whereas dimensional models differentiate by degree allowing for more nuance Koltko-Rivera (2004) and are less open to reductionism. He posits 42 dimensions for worldviews (2004, pp. 29–30).

4 Hegel, G ‘The philosophy of History’ translated Sibree, J. in ‘The Great books of the Western World’ Vol 46. Chicago: Encyclopaedia Britannica, 1952, p. 193).

5 There is no documentation to evidence whether this was a conscious decision or a more passive marginalisation, whereby it was not even considered. However, the difference between the Welsh documentation and the English is interesting.

6 ‘30% of primary schools responded to a NATRE survey in 2016 spent less than 45mins a week on RE’, CoRE 2018: 8.

7 Ofsted is the Office for Standards in Education, Children’s Services and Skills.

## References

[CR2] Aerts D., Apostel, L., De Moor, B., Hellemans, S., Maex E., Van Belle, H., & Van der Veken, J. (2007) *Worldviews. From fragmentation to integration*. Internet edition of original 1994. VUB Press. http://www.vub.ac.be/CLEA/pub/books/worldviews.pdf.

[CR1] Apostel, L., Van der Veken, J. (1991) Wereldbeelden, DNB/Pelckmans. Translated with some additions in (Aerts et al.1994).

[CR3] Barnes LP (2015). Humanism, non-religious worldviews and the future of religious education. Journal of Beliefs and Values.

[CR4] Chater M (2020). Reforming RE.

[CR5] Connor W (1994). Ethno-Nationalism: The quest for understanding.

[CR6] Cooling T (2020). Worldview in religious education: autobiographical reflections on The Commission on Religious Education in England final report. British Journal of Religious Education.

[CR7] Commission on Religious Education. (2018). *Final Report: Religion and Worldviews: The Way Forwards. A National Plan*. London: Religious Education Council of England & Wales. Retrieved from https://www.commissiononre.org.uk/wp-content/uploads/2018/09/Final-Report-of-the-Commission-on-RE.pdf.

[CR8] Copley T (2005). Indoctrination, Education and God. The struggle for the mind.

[CR9] Dewey J (1933). How we think: a restatement of the relation of reflective thinking in the education process.

[CR10] Dilthey. (1907). ‘The Types of World Views and their Unfoldment within the Metaphysical Systems’. In *Dilthey's Philosophy of existence*: *Introduction to Weltanschauungslehre*. Translation by William Kluback and Martin Weinbaum. Westport, CT: Greenwood Press, 1978, 1957.

[CR11] Dinham, A., & Shaw, M. (2015). *RE for Real: The future of teaching and learning about religion and belief*. Retrieved from https://www.gold.ac.uk/media/documents-by-section/departments/research-centres-and-units/research-units/faiths-and-civil-society/REforREal-web-b.pdf.

[CR12] Duderija A (2007). Islamic Groups and their worldviews and identities: Neo-traditional Salafis and Progressive Muslims. Arab Law Quarterly.

[CR13] Flanagan R (2019). Implementing a Ricoeurian lens to examine the impact of individuals' worldviews on subject content knowledge in RE in England: A theoretical proposition. British Journal of Religious Education.

[CR14] Freathy R, John HC (2019). Worldview and big ideas: A way forward for religious education. Nordidactica.

[CR15] Freud S (1933). Lecture XXXV: The Question of a Weltanschauung (1933). (published 1957).

[CR16] Freud, S. (1938). ‘*The basic writings of Sigmund Freud*.’ Brill, A. (trans). New York: The Modern Library.

[CR17] Griffiths DS, Winstanley D, Gabriel Y (2005). Learning shock: The trauma of return to formal learning. Management Learning.

[CR18] Hand M (2012). ‘What’s in a worldview? On Trevor Cooling’s Doing God in education’. Oxford Review of Education.

[CR19] Hinds, D. (2018). *Letter to the Very Reverend Dr John Hall*. Retrieved from https://www.religiouseducationcouncil.org.uk/wp-content/uploads/2018/12/Letter-to-The-Very-Reverend-Doctor-John-Hall-from-Rt-Hon-Damian-Hinds-MP...-1.jpg.

[CR20] Hedlund-de-Witt (2013). Worldviews and their significance for the global sustainable development Debate. Environmental Ethics.

[CR21] Hegel, G. W. F. (1807). *The Phenomenology of the spirit*. Translated by Miller, A.V (1977), introduction by Findlay, A. Oxford: Oxford University Press.

[CR22] Huddleston T (2007). Identity, diversity and citizenship: A critical review of educational resources.

[CR23] Joram E (2007). Clashing epistemologies: Aspiring teachers’, practising teachers’ and professors’ beliefs about knowledge and research in education. Teaching and Teacher Education.

[CR24] Jung, C. G. (1942). ‘Psychotherapy and a Philosophy of Life’. In ‘The Practice of Psychotherapy: essays on the Psychology of the Transference and other subjects, transited by Hull, R. pp. 76–83. Bollingen series vol 20. 2nd edition. New York: Pantheon Books 1966.

[CR25] Kant, I. (1790). ‘Critique of Judgement’. Edited and translated by Paul Guyer, University of Pennsylvania.

[CR26] Kierkegaard, S. (1838). “From the Papers of One Still Living," In *The Essential Kierkegaard*, ed. and trans. Howard V. Hong and Edna H. Hong (Princeton, New Jersey: Princeton University Press, 2000), pp.13–19.

[CR27] Kierkegaard S (1843). ‘Either/Or’, edited and translated with introduction and notes by Hong, H. and Hong, E. (1987) 2 volumes.

[CR28] Koltko-Rivera ME (2004). The psychology of worldviews. Review of General Psychology.

[CR29] Kueh R, Castelli M, Chater M (2017). Religious Education and the ‘knowledge problem’. We need to talk about Religious Education.

[CR30] Lappe F, Lappe A (2003). Hope’s Edge: the Next Diet for a Small Planet.

[CR31] Larkin S, Freathy R, Doney J, Freathy G (2020). Metacognition, Worldviews and Religious Education.

[CR32] Miedema S (2014). From religious education to worldview education and beyond: The strength of a transformative pedagogical paradigm’. Journal for the Study of Religion.

[CR33] Miedema S, Bertram-Troost GD (2015). The challenges of global citizenship for worldview education. The perspective of social sustainability. Journal of Teacher Education for Sustainability.

[CR34] Miller J, McKenna U (2011). Religion and religious education: comparing and contrasting pupils’ and teachers’ views in an English school. British Journal of Religious Education.

[CR35] Mulder A, van den Berg B (2019). Learning for life: A hermeneutical-communicative model for worldview education in light of white normativity. Religious Education.

[CR36] Naugle D (2002). Worldview: The History of a Concept.

[CR62] Nietzsche, F. (1887). *On the genealogy of morality*, Clark, M. and Swensen, A. (trans.), (1998). Indianapolis: Hackett.

[CR37] Oberg K (1960). Cultural shock: Adjustment to new cultural environments. Practical Anthropology.

[CR38] Oddrun M, Everington J (2019). Issues in the integration of religious education and worldviews education in an intercultural context. Intercultural Education.

[CR39] Ofsted. (2013). *Religious Education: realising the potential*. Retrieved from www.ofsted.gov.uk/resources/130068.

[CR40] Olsen M, Lodwick D, Dunlap R (1992). Viewing the World Ecologically.

[CR41] Polkinghorne DE (1995). Narrative configuration in qualitative analysis. International Journal of Qualitative Studies in Education.

[CR42] Prothero S (2007). Religious illiteracy: What every American needs to know –and Doesn’t.

[CR43] Riessman CK (2008). Narrative methods for the human sciences.

[CR44] Rollock N (2009). NQT Achieving Race Equality in schools training programme. Final report.

[CR45] Rorty R (1982). Consequences of pragmatism: Essays: 1972–1980.

[CR46] Samovar L, Porter R (2004). Communication between cultures.

[CR47] Schultz K, Swezey J (2013). A three-dimensional concept of worldview. Journal of Research on Christian Education.

[CR48] Smalley, P. (2018). *Response to the Commission on Religious Education’s Final Report, Religion and Worldviews: The Way Forward.* National Association of the Standing Advisory Councils on Religious Education. Retrieved from https://nasacre.org.uk/file/nasacre/1-166-response-to-core.pdf.

[CR49] Sire J (1988). The Universe next door.

[CR50] Sire J (2004). Naming the elephant. Worldview as a concept.

[CR51] Uono S, Hietanen JK (2015). Eye contact perception in the west and east: A cross-cultural study. PLoS ONE.

[CR52] Van der Kooij J, de Ruyter D, Midema S (2013). Worldview’: the meaning of the concept and the Impact on Religious Education. Religious Education.

[CR53] Valk J (2009). Knowing self and others: worldviews study at Renaissance college. Journal of Adult Theological Education.

[CR54] Valk J, Tosun A (2016). Enhancing religious education through worldview exploration. Discourse and Communication for Sustainable Education.

[CR55] Vidal, C. (2008). What is a worldview? Published in Dutch as: "Wat is een wereldbeeld?". In Hubert Van Belle and Jan Van der Veken (Eds), *Nieuwheid Denken. De Wetenschappen En Het Creatieve Aspect Van De Werkelijkheid* (pp. 71–85). Leuven: Acco*. *https://cogprints.org/6094/.

[CR56] Vroom, H. (2006). *A spectrum of worldviews: An Introduction To Philosophy Of Religion In A Pluralistic World.* Translated by Greidanus, M and A. Amsterdam: Rodopi.

[CR57] Walsh B, Middleton R (1984). The transforming vision.

[CR58] Weller M, Wolff S (2005). Autonomy, self-governance and conflict resolution.

[CR59] Welsh Government. (2020). *Curriculum document for Wales: Religion, values and ethics.* Cardiff: OGL, Crown copyright. Retrieved from https://gov.wales/sites/default/files/consultations/2020-05/consultation-document-curriculum-for-wales-religion-values-and-ethics.pdf.

[CR60] Wolters A (1985). Creation regained: Biblical basis for a reformational worldview.

[CR61] Woodhead L (2016). ‘The rise of no religion in Britain: The emergence of a new cultural majority. Journal of the British Academy..

